# Real World Evidence of Clinical Outcomes of First-Line Chemotherapy in Locally Advanced and Metastatic Pancreatic Adenocarcinoma Patients

**DOI:** 10.31557/APJCP.2026.27.1.371

**Published:** 2026-01-22

**Authors:** Nunticha Umpawan, Panotpol Termsinsuk, Uayporn Kaosombatwattana, Charuwan Akewanlop, Krittiya Korphaisarn

**Affiliations:** 1 *Division of Medical Oncology, Department of Medicine, Faculty of Medicine Siriraj Hospital, Mahidol University, Bangkok, Thailand.*; 2 *Division of Gastroenterology, Department of Medicine, Faculty of Medicine Siriraj Hospital, Mahidol University, Bangkok, Thailand.*

**Keywords:** Pancreatic ductal adenocarcinoma, Outcomes, Side effects, Survival

## Abstract

**Purpose:**

Pancreatic cancer has poor prognosis, with a five-year survival rate of approximately 10%. This study evaluated the clinical outcomes of first-line chemotherapy (CMT) for patients with locally advanced and metastatic pancreatic ductal adenocarcinoma (LA/M-PDAC).

**Patients and Methods:**

A retrospective chart review was conducted of patients with LA/M-PDAC who underwent CMT between January 2008 and December 2018. Efficacy data (objective response rate [ORR], disease control rate [DCR], progression-free survival [PFS], and overall survival [OS]) were evaluated and compared using Pearson’s chi-squared tests, Kaplan–Meier plots, and log-rank tests.

**Results:**

Of 998 patients diagnosed with LA/M-PDAC, 332 (33.3%) underwent systemic CMT. Among the treatment regimens used, gemcitabine (GEM) was most commonly administered (33.7%). The next most common therapies were (m)FOLFIRINOX and GEM plus capecitabine (GEMCAPE), accounting for 27.4% and 26.2% of cases, respectively. The ORRs were 4.5, 10.3, 23.1, and 19.4% for GEM, GEMCAPE, (m)FOLFIRINOX, and Platinum doublets (PlatD), respectively. Patients who received combination CMTs had significantly longer median PFS than those who received GEM alone (PFS = 4.93 months (mos) for GEMCAPE, 9 mos for (m)FOLFIRINOX, 9.43 mos for PlatD, and 3.87 mos for GEM). However, no significant differences were observed in the median OS rates among the four regimens. The treatment-related grade 3 or 4 adverse events were highest in the (m)FOLFIRINOX group.

**Conclusion:**

In the first-line treatment of PDAC, (m)FOLFIRINOX exhibited higher ORR and PFS than GEM or GEMCAPE. However, no survival advantage was observed in the (m)FOLFIRINOX group, suggesting an influence of subsequent therapy.

## Introduction

Pancreatic cancer is the fourth leading cause of cancer-related death worldwide [1]. Symptoms are rarely apparent in patients, and there are no specific markers to aid in the detection of early stage tumors. Unfortunately, diagnosis is often delayed until the cancer progresses to a locally advanced or metastatic stage. Less than 20% of patients are candidates for resection, and even after resection and adjuvant therapy, approximately 80% of patients relapse and ultimately die from the disease [2].

The prognosis of patients with advanced-stage pancreatic cancer is poor, with a 1-year survival rate of approximately 18% and 5-year survival rate of less than 8% [3, 1]. Additionally, pancreatic cancers exhibit a few prevalent genetic mutations, with *KRAS, CDKN2A *(encoding p16), *TP53*, and SMAD4.

Current therapeutic options are limited, and progress in drug development is impeded by the genomic, epigenetic, and metabolic complexities of pancreatic cancer. The disease involves multiple activated pathways and crosstalk, which complicate therapeutic interventions [2].

Several chemotherapeutic regimens have been developed to treat patients with advanced pancreatic cancer. In 1997, gemcitabine (GEM) became the standard first-line treatment for patients with unresectable locally advanced or metastatic pancreatic cancer (LA/M-PDAC) [4]. In 2005, erlotinib, a tyrosine kinase inhibitor of EGFR, was added to GEM as combination therapy. This resulted in a statistically significant but minimal improvement in patient outcomes compared to GEM monotherapy [5]. The combination of GEM with capecitabine (GEMCAPE) did not significantly improve overall survival (OS) compared to standard GEM treatment. However, in patients with good performance status (PS), the median OS (mOS) was significantly improved [6, 7]. In 2011, a combination of folinic acid, 5-fluorouracil, irinotecan, and oxaliplatin (m)FOLFIRINOX demonstrated significant improvements in OS compared to GEM monotherapy [8]. In 2012, a regimen of GEM and albumin-bound paclitaxel (GEM-nabP) demonstrated improved efficacy compared to GEM alone [9]. More recently, NALIRIFOX which utilizes liposomal irinotecan instead of irinotecan in (m)FOLFIRINOX showed OS benefit over GEM-nabP [10].

The chemotherapy (CMT) regimen should be based on the patient’s condition and treatment needs because of the diverse characteristics of the currently available regimens. The National Comprehensive Cancer Network guidelines recommend that patients with LA/M-PDAC and an Eastern Cooperative Oncology Group (ECOG) PS of 0 or 1 should receive first-line treatment with GEM-nabP, (m)FOLFIRINOX, or NALIRIFOX. In contrast, patients with ECOG PS 2 should receive GEM monotherapy or the best supportive care [11].

Our study compared the real-world clinical outcomes and safety of four standard regimens used as first-line treatment for LA/M-PDAC at Siriraj Hospital: GEM, GEMCAPE, (m)FOLFIRINOX, and platinum-doublets (PlatD).

## Materials and Methods

This retrospective cohort study was conducted at the Faculty of Medicine, Siriraj Hospital, Bangkok, Thailand between January 2008 and December 2018. This study included patients with LA/M-PDAC who had received treatment at our center. Patients were excluded if they had missing data, no histopathological data, or did not receive treatment or follow-up at our center.

The Siriraj Institutional Review Board approved the study protocol (approval number: Si 285/2020). In this retrospective study, patient data remained anonymous and patient health and well-being were unaffected. Therefore, the need to obtain written informed consent from study participants was waived.

The primary objective of this study was to evaluate the effects of different CMT regimens on progression-free survival (PFS) in patients with LA/M-PDAC. The secondary objective was to assess the impact of CMT regimens on the objective response rate (ORR), disease control rate (DCR), OS, and toxicities.

### Clinical characteristics

Demographic and clinical data were collected from the electronic medical records of the patients with LA/M-PDAC. The data included age, sex, ECOG PS performance status, tumor location, disease extent, differentiated histology, carbohydrate antigen 19-9 (CA 19-9) levels, and biliary stent insertion. We also collected details on the CMT regimen used, date of LA/M-PDAC diagnosis, date of disease recurrence, dates of CMT initiation and cessation, date of last follow-up, and date of death.

Disease staging was determined using the American Joint Committee on Cancer TNM Staging Classification System for Pancreatic Cancer (8th edition) [12]. The response rate was evaluated using the Response Evaluation Criteria in Solid Tumors (RECIST) version 1.1.

### Statistical analysis

The study calculated PFS from the date of starting CMT to the date of disease progression or death (whichever occurred first). The OS was calculated from the date of CMT initiation to the date of death from any cause.

This study described patient demographics and clinical characteristics using summary statistics. Median and range values are reported for continuous variables, whereas frequency and percentage values are presented for categorical variables. Pearson’s chi-square test or Fisher’s exact test was used to evaluate multiple comparisons between the CMT regimens and clinicopathological variables.

Patient survival outcomes were analyzed using the Kaplan–Meier method, and differences between survival curves were determined using the log-rank test. Cox regression analysis was performed to estimate the 95% confidence intervals (CIs) for PFS. A probability (P) value of ≤ 0.05 indicated statistical significance. Statistical analyses were performed using the IBM SPSS Statistics version 21 (IBM Corp., Armonk, NY, USA).

## Results

**Figure 1:**
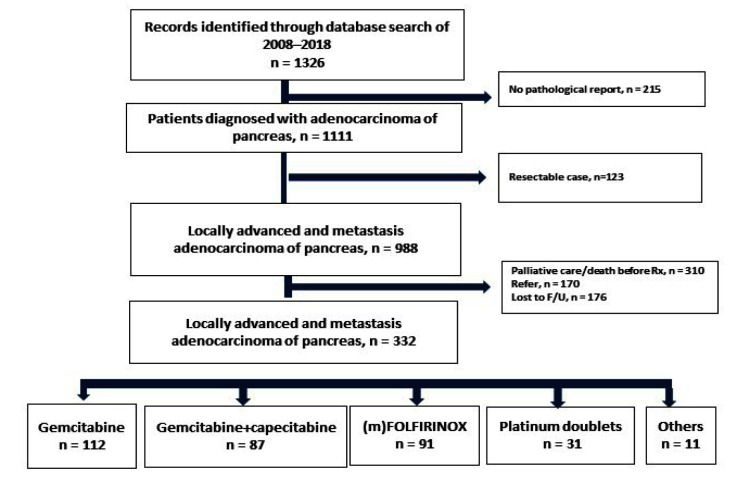
CONSORT Diagram of Patient Enrollment

Between January 2008 and December 2018, 988 patients were diagnosed with LA/M-PDAC at our center. Of these, 332 (33.3%) patients received systemic CMT. A Consolidated Standards of Reporting Trials diagram illustrating patient enrollment is shown in Figure 1.

### Patient characteristics

The analysis included 321 patients who received GEM, GEMCAPE, (m)FOLFIRINOX, or PlatD regimens. Most patients received GEM monotherapy (34.9%); (m)FOLFIRINOX (28.3%), GEMCAPE (27.1%), and PlatD (9.7%) were the most commonly used combination regimens.

The median age of the patients was 63 years (range: 30-87), and the female-to-male ratio was 1.24:1. Of the total patients, 31.2% were diagnosed with LA-PDAC, and 68.8% were diagnosed with M-PDAC. Most patients had an ECOG performance status (PS) of 0–1 (90.3%). The median baseline carbohydrate antigen 19-9 (CA19-9) level was 381.9 U/mL. Biliary stent insertion was performed in 25% of the patients, with 70.9% receiving metallic stents and 29.1% receiving plastic stents. Table 1 presents the demographic and clinical characteristics of the patients according to CMT regimen.

### Efficacy

#### Clinical response

The tumor response data are presented in Table 2. The ORRs for patients who received the GEM, GEMCAPE, (m)FOLFIRINOX, and PlatD regimens were 4.5%, 10.3%, 23.1%, and 19.4%, respectively (P < 0.001).

Only one patient in the PlatD regimen achieved CR. In the GEM group, 21 (18.8%) patients did not undergo assessment after the baseline visit. Similarly, eight patients (9.2%) in the GEMCAPE group, 11 (12.1%) in the (m)FOLFIRINOX group, and three (9.7%) in the PlatD group did not undergo assessment after their baseline visits.

### Subsequent treatment

Data on subsequent therapies are detailed in Table 3. XELOX or FOLFOX regimen was the most common second-line CMT after GEM (15.2%) and GEMCAPE (34.5%). After (m)FOLFIRINOX, GEMCAPE was the most commonly used second-line regimen (22%).

Of the 321 patients in the study, 63% (202/321) did not receive any treatment after the first-line CMT. Only 7% of the patients received third-line therapy.

### Survival

The median follow-up duration was 40 months. As of the data cutoff date (March 15, 2020), 11.2% (26/321) of the patients were still alive, whereas 88.8% (285/321) died.

Univariate analysis of PFS was performed using established prognostic factors (Table 4). The following factors were associated with a significantly better PFS:

• ECOG PS of 0–1 (hazard ratio [HR] = 0.43; P < 0.001)

• LA-PDAC (HR = 0.72; P = 0.02)

• normalized CA19-9 level (HR = 0.66; P = 0.03)

• best response without progressive disease (stable disease [SD] vs. progressive disease [PD]: HR = 0.16, P < 0.0001; CR or PR vs. PD: HR = 0.11, P < 0.0001).

Compared to GEM, all combination CMT regimens had significantly longer PFS. (GEMCAPE vs GEM: 4.93 vs 3.87 months, with HR = 0.72 and P = 0.04; (m)FOLFIRINOX vs GEM: 9.0 vs 3.87 months, with HR = 0.39 and P < 0.001; PlatD vs GEM: 9.43 vs 3.87 months, with HR = 0.42 and P < 0.001).

Multivariate Cox proportional hazard regression analysis of PFS was performed using these factors (Table 4). The following factors remained associated with better PFS.

• combination CMT regimens (GEMCAPE: HR = 0.66, P = 0.01; (m)FOLFIRINOX: HR = 0.38, P < 0.001; PlatD: HR = 0.46, P < 0.003).

• tumors located in the body of the pancreas (HR = 0.58; P = 0.03)

• normalized CA19-9 level (HR = 0.61; P = 0.02)

• best response without PD (SD vs. PD: HR = 0.16, P < 0.0001; CR or PR vs. PD: HR = 0.12, P < 0.0001).

In the OS analysis, the following factors were found to be significantly associated with better OS:

• ECOG PS 0–1 (HR = 0.54; P = 0.002)

• normalized CA19-9 (HR = 0.57; P = 0.001)

• CA19-9 < 59 x UNL (HR = 0.54; P < 0.0001)

• best response with no PD (SD vs. PD: HR = 0.46, P < 0.0001; CR/PR vs. PD: HR = 0.27, P < 0.0001).

• received subsequent therapy (HR = 0.51; P < 0.001)

All CMT regimens had significantly longer OS than GEM (GEMPCAPE: HR = 0.75, P = 0.05; (m)FOLFIRINOX: HR = 0.64, P = 0.004; PlatD: HR = 0.64, P = 0.04).

**Figure 2:**
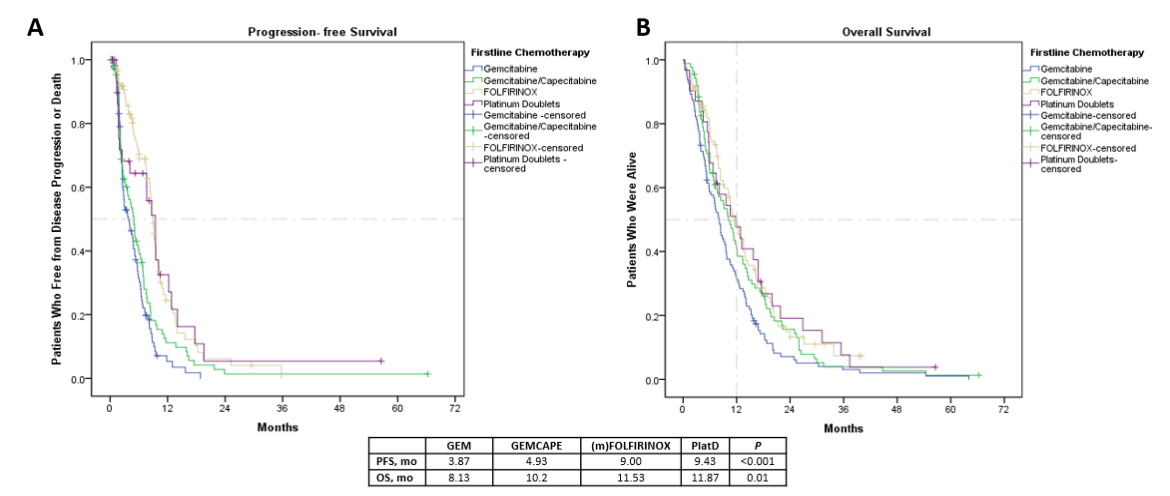
PFS and OS According to Chemotherapy Regimen

Table 5 and Figure 2 present the detailed results of the multivariate Cox proportional hazards regression analysis of the OS. The analysis revealed that the following factors were associated with a better OS:

• normalized CA19-9 (HR = 0.53; P = 0.001)

• CA19-9 < 59 x UNL (HR = 0.54; P < 0.0001)

• Best response with no PD (SD vs. PD: HR = 0.45, P < 0.0001; CR/PR vs. PD: HR = 0.26, P < 0.0001).

• received subsequent therapy (HR = 0.51; P < 0.001)

The one-year survival rates for GEM, GEMCAPE, (m)FOLFIRINOX, and PlatD were 22%, 31%, 36%, and 41%, respectively (Table 2).

A comparison of the two most popular regimens showed that (m)FOLFIRINOX had a significantly better PFS than GEMCAPE (9 vs 4.93 months; HR = 0.53; P < 0.0001), which was confirmed by multivariate analysis (HR = 0.62; P = 0.02; Supplementary Table 1). However, there were no significant differences in the OS between the two regimens in the univariate (HR = 0.85; P = 0.33) and multivariate analyses (HR = 1.07; P = 0.73; Supplementary Table 2; Supplementary Figure 1).

### Safety

The incidence of treatment-related grade 3 or 4 adverse events was highest in the (m)FOLFIRINOX group, followed by the GEMCAPE, PlatD, and GEM groups. The febrile neutropenia rate in the (m)FOLFIRINOX group was 5.6 %. The incidence of grade 3/4 anemia was significantly higher in the (m)FOLFIRINOX and PlatD groups, whereas the incidence of grade 3/4 palmar-plantar erythrodysesthesia was significantly higher in the GEMCAPE group (8.1%). The incidence of grade 3/4 diarrhea was 11.2%, 3.5%, and 2.7% in the (m)FOLFIRINOX, GEMCAPE, and GEM group, respectively, with a significant difference between the groups (P = 0.02). CMT-related adverse events leading to discontinuation occurred in 31.7% and 23.8% of patients in the (m)FOLFIRINOX and GEMCAPE groups, respectively. All patients who received (m)FOLFIRINOX also received filgrastim. Supplementary Table 3 summarizes the treatment-related adverse events in all treatment groups.

## Discussion

This study compared the effectiveness of combination CMT regimens (GEMCAPE, (m)FOLFIRINOX, and PlatD) with GEM monotherapy as first-line treatment for patients with LA/M-PDCA. The results showed that combination CMTs had a longer median PFS than GEM monotherapy, with the highest ORR in the (m)FOLFIRINOX group. However, there was no significant difference in OS among the first-line CMT regimens. Patients who received combination CMTs experienced more grade 3 or 4 adverse events than those who received GEM monotherapy.

Systemic CMT is the standard therapy for LA/M-PDAC and has shown improved survival compared with the best supportive care in randomized trials [13]. Both (m)FOLFIRINOX [8] and GEM-nabP [9] have demonstrated survival benefits over GEM monotherapy, making them the preferred first-line treatment. However, (m)FOLFIRINOX is not recommended for patients aged > 75 years or those with an ECOG PS of 2. Consequently, (m)FOLFIRINOX is the most commonly used regimen; however, GEM-nabP is preferred for older patients and those with a lower PS. Recent data from systematic reviews and meta-analyses indicate that (m)FOLFIRINOX provides comparable survival benefits with fewer adverse events than the conventional dosage [14]. More recently, NALIRIFOX demonstrated improvements in OS compared with GEM-nabP [10]. These findings support the use of either NALIRIFOX or (m)FOLFIRINOX regimen as the preferred first-line treatment for metastatic pancreatic cancer in patients able to tolerate a three-drug regimen.

Although GEMCAPE has not been found to improve OS compared to GEM in randomized studies, it has shown a significant improvement in mOS in patients with good ECOG PS [6, 7]. A meta-analysis [15] also found that GEMCAPE significantly improved OS (HR = 0.87; P = 0.03) and ORR (ORR = 0.66; P = 0.03) compared to GEM alone. A recent systematic review and meta-analysis of randomized controlled trials [16] involving 1879 patients found that the GEM had a statistically significant increase in HR for OS (HR = 1.15; 95% CI: 1.037–1.276; P = 0.008) and PFS (HR = 1.211; 95% CI: 1.09–1.344; P = 0.860) compared to the GEMCAPE group. In clinical practice, GEMCAPE may be considered an alternative to single-agent GEM for patients with good PS.

The treatment options for patients with LA/M-PDAC at Siriraj Hospital were limited as GEM-nabP was not available at that time. Consequently, patients were offered alternative CMT options such as (m)FOLFIRINOX, mFOLFIRINIOX, GEMCAPE, or single-agent GEM. Patients with good PS were often prescribed GEMCAPE because of its easy administration, tolerability, lower incidence of side effects, and lower cost than (m)FOLFIRINOX. However, the efficacy and safety of these two regimens have not been directly compared in clinical trials; therefore, a regimen that is more effective remains to be determined.

The baseline characteristics of the patients enrolled in this study were similar to those of previously published data on first-line treatment, except for the slightly higher proportion of patients with an ECOG PS score of 2. However, none of the patients in our study who received (m)FOLFIRINOX had an ECOG PS score of 2. Treatment options were selected based on individual patient factors and preferences. The ORR and DCR differed significantly among the treatment groups. The highest ORR rate was observed in the (m)FOLFIRINOX group (23.1%), followed by the PlatD (19.4%) and GEMCAPE therapy (10.3%) groups, and the lowest rate was observed in the GEM group (4.5%; Table 2). These ORR rates were slightly lower than those previously reported (ORR = 32% for (m)FOLFIRINOX, 19% for GEMCAPE, and 10% for GEM) [6, 8, 17, 7]. However, caution is necessary when interpreting these results because of the differences in patient characteristics between our study and the randomized studies. Nevertheless, the high ORR rate in the (m)FOLFIRINOX group is consistent with previous randomized studies [8]. 

Our study findings showed that the median PFS was significantly longer for all combination CMT regimens than for GEM monotherapy, consistent with previous phase III trials [8, 9, 16]. However, there was no significant difference in OS between the treatment groups. This finding could be due to the influence of subsequent therapy, which may have obscured the potential difference in the mOS.

When compared with real-world data from an Asian population, a nationwide, population-based study conducted in Korea between 2012 and 2019, which included 8,652 patients, reported a median mOS of 11 months with (m)FOLFIRINOX and 6 months with GEM monotherapy. These findings are consistent with the OS outcomes observed in our cohort for the (m)FOLFIRINOX group, although the OS for the gemcitabine group was slightly higher in the Korean study [18].

Notably, 60% of the patients in our study did not receive treatment beyond first-line therapy, possibly indicating a decline in their ECOG PS. This proportion is consistent with that of Smyth et al., who reported that only 40% of patients could receive further therapy after first-line treatment [19].

In a systematic review and indirect comparison conducted by Kharat et al. [20], the effectiveness of the two most commonly used regimens, GEMCAPE and (m)FOLFIRINOX, was compared. Their study reported that (m)FOLFIRINOX had a significantly better OS (HR = 0.71; 95% CI: 0.60–0.85) and PFS (HR = 0.65; 95% CI: 0.57–0.74) than GEMCAPE. Our study also found an improvement in PFS with (m)FOLFIRINOX compared to GEMCAPE (HR = 0.53; 95% CI: 0.38–0.75; P < 0.0001). However, there was no significant difference in OS. Notably, more patients in the GEMCAPE group received second- and later-line CMT (46%) than those in the (m)FOLFIRINOX group (36%). This disparity in the proportion of patients receiving subsequent therapy may explain the lack of a significant difference in the mOS between the two regimens.

Patients with ECOG PS 2, elevated CA19-9 levels ≥ 59 × ULN, and tumors located in the body of the pancreas have been shown to have an increased risk of mortality compared to those with ECOG PS 0–1, normal CA19-9 levels, and tumors located in the head or tail [21-25]. Consistent with these findings, our data demonstrated that ECOG PS 2 and elevated CA19-9 levels ≥ 59 × ULN were associated with an increased mortality risk. However, we did not observe a significant difference in OS based on the tumor location.

Regarding side effects, our study found that the combination group had a higher incidence of treatment-related grade 3 or 4 adverse events. Specifically, (m)FOLFIRINOX was associated with a higher incidence of grade 3 or 4 thrombocytopenia, diarrhea, and sensory neuropathy, while GEMCAPE was associated with a higher rate of palmar-plantar erythrodysesthesia. These results are consistent with those of previous randomized studies on first-line treatment [6-8]. Additionally, the (m)FOLFIRINOX group in the real-world Korean study demonstrated a significantly higher hazard ratio (HR) for febrile neutropenia (HR: 2.285; 95% CI: 1.864–2.802) and hospitalization (HR: 1.16; 95% CI: 1.056–1.274), which is similar to our results [18]. The incidence of febrile neutropenia in our study was 5.6%, comparable to the rate of 5.4% reported in the (m)FOLFIRINOX group in a study by Conroy et al. [8]. In their study, filgrastim was not recommended as primary prophylaxis, whereas in our study, filgrastim was administered to all patients receiving (m)FOLFIRINOX. This difference in regimens indicates that the FN rate in our patients might have been higher if no filgrastim prophylaxis had been used.

### Study strengths and limitations

The main strength of this study was that it focused on the effect of multiple first-line CMT regimens in patients with LA/M-PDAC, reflecting real-world practice. In pancreatic cancer, real-world data offers valuable insights into treatment outcomes in routine clinical settings, capturing patient populations and disease complexities often underrepresented in clinical trials. However, this study had some limitations that may have influenced the outcomes. First, incomplete or missing data and damaged documents may have affected the results. Second, confounding factors were identified through the limitations of this single-center study, including a higher risk of bias and larger treatment effect. Finally, owing to the limited size of our cohort, the statistical power of the analysis of significant associations in this population may have been insufficient. Therefore, caution should be exercised when interpreting the results of subgroup analyses. Nonetheless, our study is one of the few real-life studies to specifically address the effects of different CMT regimens on the clinical outcomes of patients with LARC.

In conclusions, this study revealed that (m)FOLFIRINOX had a higher ORR, DCR, and PFS than GEM or GEMCAPE. However, no survival benefit was observed in the (m)FOLFIRINOX group, possibly because of subsequent therapies. Although GEMCAPE had a similar OS rate compared to (m)FOLFIRINOX, it had a better safety profile. Thus, it may be considered as a first-line treatment in patients with ECOG PS 0–1 who do not require hospitalization or have a high response rate. Further randomized studies are needed to compare the efficacies of GEMCAPE and (m)FOLFIRINOX.

**Table 1:** Patient and Tumor Characteristics (N=321)

Characteristic	(n =321)	Gemcitabine (n = 112)	Gemcitabine & capecitabine (n = 87)	(m)FOLFIRINOX (n = 91)	Platinum doublets (n = 31)	P*
Age at diagnosis (yr)						
Median (range)	63 (30-87)	63 (38-87)	65 (38-86)	62 (53-69)	66 (30-79)	
Distribution — no. (%)						
<=75 yr	293 (91.3)	97 (86.6)	81 (93.1)	88 (96.7)	27 (87.1)	0.06
>75 yr	28 (8.7)	15 (13.4)	6 (6.9)	3 (3.3)	4 (12.9)	
Sex — no. (%)						0.72
Female	178 (55.5)	65 (58)	47 (54)	47 (51.6)	19(61.3)	
Male	143 (44.5)	47 (42)	40 (46)	44 (48.4)	12 (38.7)	
ECOG — no. (%)					<0.0001	
0	35 (10.9)	8 (7.1)	9 (10.3)	17 (18.7)	1 (3.2)	
1	255 (79.4)	80 (71.4)	72 (82.8)	74 (81.3)	29 (93.5)	
2	31 (9.7)	24 (21.4)	6 (6.9)	0 (0)	1 (3.2)	
Tumor location — no. (%)						0.59
Head	180 (56.1)	69 (61.6)	49 (56.3)	46 (50.5)	16 (51.6)	
Body	104 (32.4)	33 (29.5)	28 (32.2)	34 (37.4)	9 (29)	
Tail	37 (11.5)	10 (8.9)	10 (11.5)	11 (12.1)	6 (19.4)	
Extent of disease — no. (%)						0.74
Locally advanced unresectable	100 (31.2)	33(29.5)	27 (31)	32 (35.2)	8 (25.8)	
Metastatic	221 (68.8)	79(70.5)	60 (69)	59 (64.8)	23 (74.2)	
Histology — no. (%)						0.53
Well differentiated	18 (5.6)	7 (6.3)	3 (3.4)	7 (7.7)	1 (3.2)	
Moderately differentiated	143 (44.5)	47 (42)	43 (49.4)	36 (39.6)	17 (54.8)	
Poorly differentiated	27 (8.4)	11 (9.8)	4 (4.6)	11 (12.1)	1 (3.2)	
Unknown	133(41.4)	47 (42)	37 (42.5)	37 (40.7)	12 (38.7)	
Biliary stent — no. (%)						0.66
No	242 (75.4)	84 (75)	62 (71.3)	71 (78)	25 (80.6)	
Yes	79 (24.6)	28 (25)	25 (28.7)	20 (22)	6 (19.4)	
Plastic	23 (29.1)	7 (25)	6 (24)	8 (40)	2 (33.3)	
Metallic	56 (70.9)	21 (75)	19 (76)	12 (60)	4 (66.7)	
Level of carbohydrate antigen 19-9 no./total no. (%)						0.1
Normal†	68 (21.2)	27 (24.1)	10 (11.5)	25 (27.5)	6 (19.4)	
Elevated, <59 x ULN	152 (47.4)	55 (49.1)	43 (49.4)	39 (42.9)	15 (48.4)	
Elevated, ≥59 x ULN	91 (28.3)	27 (24.1)	32 (36.8)	25 (27.5)	7 (22.6)	
Unknown	10 (3.1)	3 (2.7)	2 (2.3)	2 (2.2)	3 (9.7)	
CA19-9 median (range) U/mL	381.9 (40.95-2910)	302.20 (38.17-1859)	906.30 (85.02-6705)	233.70 (30.76-2398)	285.35 (53.72-2197)	

†The normal range was 0 to 35 U per milliliter; * Statistically significant; P values <0.05 are highlighted in bold text; ** Platinum doublets, including cisplatin plus gemcitabine, cisplatin plus 5FU, gemcitabine plus oxaliplatin; Abbreviations: ULN, upper limits of normal

**Table 2:** Clinical Outcomes

Efficacy variable	Gemcitabine (n = 112)	Gemcitabine & capecitabine (n = 87)	(m)FOLFIRINOX (n = 91)	Platinum doublets (n = 31)	P*
1-yr OS (%)	22	31	36	41	
mOS, mo (95% CI)	8.13 (6.81-9.46)	10.2 (7.44-12.96)	11.53 (9.50-13.57)	11.87 (5.64-18.09)	0.01
mPFS, mo (95% CI)	3.87 (2.39-5.33)	4.93 (3.76-6.08)	9 (8.29-9.71)	9.43 (6.93-11.93)	<0.0001
ORR, n (%)	5 (4.5)	9 (10.3)	21 (23.1)	6 (19.4)	<0.0001
DCR‡, n (%)	43 (38.4)	46 (52.9)	67 (73.6)	17 (54.8)	<0.0001
Detail of tumor response, n (%)					<0.0001
Complete response	0 (0)	0 (0)	0 (0)	1 (3.2)	
Partial response	5 (4.5)	9 (10.3)	21 (23.1)	5 (16.1)	
Stable disease	38 (33.9)	37 (42.5)	46 (50.5)	11 (35.5)	
Progressive disease	48 (42.9)	33 (37.9)	13 (14.3)	11 (35.5)	
Could not be evaluated‡	21 (18.8)	8 (9.2)	11 (12.1)	3 (9.7)	

‡ Included are 21 patients (18.8%) in gemcitabine group, 8 (9.2%) in gemcitabine & capecitabine, 11 (12.1) in (m)FOLFIRINOX, and 3 (9.7%) in the platinum doublets group who were not assessed after the baseline visit; * Statistically significant; P values <0.05 are highlighted in bold text; Abbreviations: PFS, progression-free survival; OS, overall survival; ORR, objective response rate; DCR, disease control rate

**Table 3:** Subsequent Treatment

	(n =321)	Gemcitabine (n = 112)	Gemcitabine & capecitabine (n = 87)	(m)FOLFIRINOX (n = 91)	Platinum doublets (n = 31)

Second-line treatment — no. (%)					
No	202 (62.9)	77 (68.8)	47 (54)	58 (63.7)	20 (64.5)
Gemcitabine	1 (0.3)	0	0	0	1 (3.2)
5FU or capecitabine or TS1	12 (3.7)	12 (10.7)	0	0	0
Irinotecan	3 (0.9)	0	2 (2.3)	0	1 (3.2)
FOLFOX/XELOX	48 (15)	17 (15.2)	30 (34.5)	0	1 (3.2)
FOLFIRI/XELIRI	10 (3.1)	0	3 (3.4)	1 (1.1)	6 (19.4)
(m)FOLFIRINOX	2 (0.6)	1 (0.9)	1 (1.1)	0	0
Gemcitabine+ capecitabine	26 (8.1)	3 (2.7)	2 (2.3)	20 (22)	1 (3.2)
Gemcitabine+ nab-paclitaxel	8 (2.5)	0	2 (2.3)	6 (6.6)	0
Gemcitabine+ erlotinib	2 (0.6)	0	0	2 (2.2)	0
Gemcitabine+ cisplatin	3 (0.9)	0	0	3 (3.3)	0
Carboplatin+ 5FU	1 (0.3)	1 (0.9)	0	0	0
Docetaxel/paclitaxel	2 (0.6)	0	0	1 (1.1)	1 (3.2)
Pembrolizumab	1 (0.3)	1 (0.9)	0	0	0
Third-line treatment — no. (%)					
No	298 (92.8)	107 (95.5)	73 (83.9)	87 (95.6)	31 (100)
5FU or capecitabine or TS1	2 (0.6)	0	2 (2.3)	0	0
Doxorubicin	1 (0.3)	0	1 (1.1)	0	0
FOLFOX/XELOX	2 (0.6)	1 (0.9)	1 (1.1)	0	0
FOLFIRI/XELIRI	12 (3.7)	3 (2.7)	8 (9.2)	1 (1.1)	0
(m)FOLFIRINOX	1 (0.3)	1 (0.9)	0	0	0
Gemcitabine+ capecitabine	1 (0.3)	0	1 (1.1)	0	0
Gemcitabine+ nab-paclitaxel	2 (0.6)	0	0	2 (2.2)	0
Docetaxel/Cisplatin/5FU	1 (0.3)	0	1 (1.1)	0	0
Atezolizumab	1 (0.3)	0	0	1 (1.1)	0

**Table 4:** Univariate & multivariate analysis of PFS according to chemotherapy regimen (n=321)

Variable	Total n/	Univariate analysis				Multivariate analysis		
	events							
		mPFS (mo)	HR	95% CI	P	HR	95% CI	P*
First-line systemic chemotherapy (n=321)								
Gemcitabine	112/88	3.87	Ref			Ref		
Gemcitabine & capecitabine	87/76	4.93	0.72	0.53-0.99	0.04	0.66	0.47-0.92	0.01
(m)FOLFIRINOX	91/62	9	0.39	0.28-0.55	<0.0001	0.38	0.27-0.55	<0.0001
Platinum doublets	31/22	9.43	0.42	0.26-0.67	<0.0001	0.46	0.28-0.76	0.003
ECOG (n=321)								
0-1	290/226	6.1	0.43	0.27-0.67	<0.0001	0.68	0.42-1.09	0.11
2	31/22	3.6	Ref			Ref		
Tumor location (n=321)								
Head	180/150	5.57	0.77	0.51-1.15	0.2	0.69	0.44-1.08	0.1
Body	104/70	6.53	0.7	0.45-1.08	0.11	0.58	0.36-0.95	0.03
Tail	37/28	4.83	Ref			Ref		
Extent of disease (n=321)								
Locally advanced unresectable	100/74	7.23	0.72	0.55-0.95	0.02	1.06	0.78-1.44	0.72
Metastatic	221/174	5.13	Ref			Ref		
Level of carbohydrate antigen 19-9 (n=311)								
Normal†	68/53	7.57	0.66	0.46-0.95	0.03	0.61	0.41-0.91	0.02
Elevated, <59 x ULN	152/125	5.57	0.8	0.59-1.09	0.15	0.79	0.57-1.08	0.14
Elevated, ≥59 x ULN	91/63	4.8	Ref			Ref		
Biliary stent (n=321)								
No	242/181	6	0.9	0.68-1.19	0.46			
Yes	79/67	4.97	Ref					
Best response (n=278)								
SD	132/105	7.93	0.16	0.12-0.22	<0.0001	0.16	0.11-0.21	<0.0001
CR/PR	41/35	9.13	0.11	0.07-0.17	<0.0001	0.12	0.08-0.19	<0.0001
PD	105/105	2.07	Ref			Ref		

* Statistically significant; P value <0.05; Abbreviations: ULN, upper limits of normal; PFS, Progression-free survival; OS, overall survival; SD, stable disease; CR, complete response; PR, partial response; PD, progressive disease

**Table 5:** Univariate & Multivariate Analysis of OS According to Chemotherapy Regimen (n=321)

Variable	Total n/ events	Univariate analysis				Multivariate analysis		
		mOS (mo)	HR	95% CI	P	HR	95% CI	P*
First-line systemic chemotherapy (n=321)								
Gemcitabine	112/108	8.13	Ref			Ref		
Gemcitabine & capecitabine	87/80	10.2	0.75	0.56-1.00	0.05	1.08	0.77-1.51	0.65
(m)FOLFIRINOX	91/69	11.53	0.64	0.47-0.87	0.004	1.02	0.71-1.47	0.92
Platinum doublets	31/28	11.87	0.64	0.42-0.97	0.04	0.68	0.42-1.09	0.11
ECOG (n=321)								
0-1	290/256	10.5	0.54	0.36-0.79	0.002	0.76	0.46-1.26	0.29
2	31/29	5.23	Ref			Ref		
Tumor location (n=321)								
Head	180/161	10	1	0.69-1.43	0.98	1.19	0.75-1.89	0.46
Body	104/88	9.57	1.15	0.78-1.70	0.5	1.35	0.83-2.22	0.23
Tail	37/36	10.57	Ref			Ref		
Extent of disease (n=321)								
Locally advanced unresectable	100/86	10.5	0.87	0.68-1.12	0.28	0.82	0.62-1.09	0.17
Metastatic	221/199	9.57	Ref			Ref		
Level of carbohydrate antigen 19-9 (n=311)								
Normal†	68/61	10.6	0.57	0.41-0.79	0.001	0.53	0.36-0.77	0.001
Elevated, <59 x ULN	152/130	11.33	0.54	0.41-0.72	<0.0001	0.54	0.39-0.74	<0.0001
Elevated, ≥59 x ULN	91/84	5.97	Ref			Ref		
Biliary stent (n=321)								
No	242/212	9.9	0.87	0.66-1.13	0.29			
Yes	79/73	9.07	Ref					
Best response (n=278)								
SD	132/112	13.37	0.46	0.35-0.60	<0.0001	0.45	0.34-0.60	<0.0001
CR/PR	41/32	16.8	0.27	0.18-0.40	<0.0001	0.26	0.17-0.39	<0.0001
PD	105/100	5.8	Ref			Ref		
Subsequent line (n=321)								
No	202/176	7.17	Ref			Ref		
Yes	119/109	14.47	0.51	0.40-0.65	<0.0001	0.51	0.39-0.67	<0.0001

* Statistically significant; P value <0.05; Abbreviations: ULN, upper limits of normal; PFS, Progression-free survival; OS, overall survival; SD, stable disease; CR, complete response; PR, partial response; PD, progressive disease

## Author Contribution Statement

All authors helped to perform the research; NU collected the data, analyzed the data and drafted the manuscript; PT collected the data, reviewed and edited manuscript; UK analyzed the data, reviewed and edited manuscript; CA co-supervised the field activities, analyzed the data, reviewed and edited manuscript; KK designed the study, supervised the study, collected the data, analyzed the data, reviewed and edited the manuscript, and approved the final version..
