# Supplementary figures and images for: Real World Evidence of Clinical Outcomes of First-Line Chemotherapy in Locally Advanced and Metastatic Pancreatic Adenocarcinoma Patients
Source: Asian Pac J Cancer Prev. 2026 Jan 22;27(1):371–80. doi: 10.31557/APJCP.2026.27.1.371 (PMC13418030; doi:10.31557/APJCP.2026.27.1.371)

## Supplementary Figure 1. PFS and OS according to chemotherapy regimen

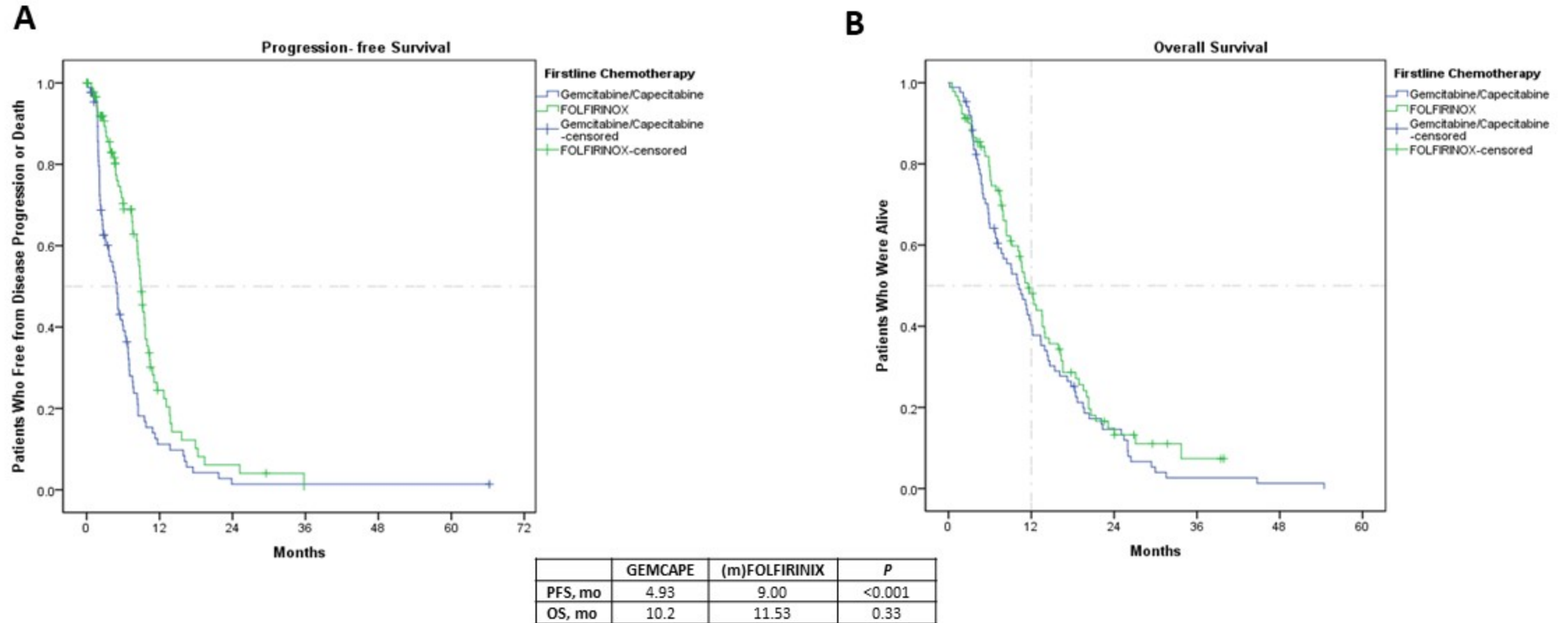

Supplement: Figure S1 [file APJCP-27-1-371-s001.pdf]
