# Supplementary material for: Real World Evidence of Clinical Outcomes of First-Line Chemotherapy in Locally Advanced and Metastatic Pancreatic Adenocarcinoma Patients
Source: Asian Pac J Cancer Prev. 2026 Jan 22;27(1):371–80. doi: 10.31557/APJCP.2026.27.1.371 (PMC13418030; doi:10.31557/APJCP.2026.27.1.371)
Supplement: Tables S1-S3 [file APJCP-27-1-371-s002.pdf]

Supplementary Table1. Univariate & multivariate analysis of PFS in gemcitabine & capecitabine vs (m)FOLFIRINOX

Abbreviations: dMMR, deficient MMR; proficient MMR

Variable	Total n/ events	Univariate analysis				Multivariate analysis		
		mPFS (mo)	HR	95% CI	*P*	HR	95% CI	*P**
**First-line systemic chemotherapy** (n=178)								
Gemcitabine & capecitabine	87/76	**4.93**	Ref			Ref		
(m)FOLFIRINOX	91/62	9.00	0.53	0.38-0.75	**<0.0001**	0.62	0.42-0.91	**0.02**
**ECOG (n=178)**								
0-1	172/132	**7.37**	0.19	0.08-0.44	**<0.0001**	0.52	0.21-1.29	0.16
2	6/6	2.13	Ref			Ref		
**Tumor location** (n = 178)								
Head	95/80	6.80	Ref			Ref		
Body	62/41	7.07	0.97	0.67-1.42	0.88	0.86	0.58-1.26	0.43
Tail	21/17	7.37	1.50	0.88-2.55	0.14	1.84	1.04-3.26	**0.04**
**Extent of disease (n=178)**								
Locally advanced unresectable	59/43	8.8	0.60	0.42-0.87	**0.01**	0.87	0.57-1.32	0.51
Metastatic	119/95	6.0	Ref			Ref		
**Level of carbohydrate antigen 19-9 (n=174)**								
Normal†	35/25	9.13	0.52	0.32-0.86	**0.01**	0.49	0.28-0.84	**0.01**
Elevated, <59 x ULN	82/66	6.53	0.62	0.42-0.91	**0.02**	0.69	0.46-1.04	0.07
Elevated, ≥59 x ULN	57/44	5.10	Ref			Ref		
**Biliary stent (n=178)**								
No	133/97	7.37	0.84	0.58-1.20	0.33			
Yes	45/41	5.73	Ref					
**Best response (n=159)**								
SD	83/65	8.23	0.23	0.16-0.34	**<0.0001**	0.22	0.15-0.34	**<0.0001**
CR/PR	30/25	9.13	0.17	0.10-0.28	**<0.0001**	0.16	0.09-0.27	**<0.0001**
PD	46/46	2.07	Ref			Ref		

* Statistically significant; *P* value <0.05

Abbreviations: ULN, upper limits of normal; PFS, Progression-free survival; OS, overall survival; SD, stable disease; CR, complete response; PR, partial response; PD, progressive disease

Supplementary Table 2. Univariate & multivariate analysis of OS in gemcitabine & capecitabine vs (m)FOLFIRINOX

Variable	Total n/ events	Univariate analysis				Multivariate analysis		
		mOS (mo)	HR	95% CI	*P*	HR	95% CI	*P**
**First-line systemic chemotherapy** (n=178)								
Gemcitabine & capecitabine	87/80	**10.2**	Ref			Ref		
(m)FOLFIRINOX	91/69	11.53	0.85	0.62-1.18	0.33	1.07	0.72-1.59	0.73
**ECOG** (n=178)								
0-1	172/143	**11.17**	0.34	0.15-0.78	**0.01**	0.31	0.13-0.77	**0.01**
2	6/6	4.07	Ref			Ref		
**Tumor location** (n=178)								
Head	95/81	10.73	Ref			Ref		
Body	62/48	10.50	1.34	0.94-1.93	0.11	1.60	1.06-2.41	**0.03**
Tail	21/20	11.17	1.14	0.70-1.87	0.59	1.29	0.71-2.33	0.41
**Extent of disease** (n=178)								
Locally advanced unresectable	59/47	11.7	0.77	0.54-1.09	0.14	0.69	0.45-1.06	0.09
Metastatic	119/102	10.0	Ref			Ref		
**Level of carbohydrate antigen 19-9** (n=174)								
Normal†	35/30	12.27	0.71	0.45-1.12	0.14	0.58	0.35-0.97	**0.04**
Elevated, <59 x ULN	82/65	11.70	0.54	0.37-0.78	**0.001**	0.50	0.32-0.78	**0.002**
Elevated, ≥59 x ULN	57/50	7.93	Ref			Ref		
**Biliary stent** (n=178)								
No	133/108	10.60	Ref					
Yes	45/41	13.37	0.93	0.45-1.34	0.69			
**Best response** (n=159)								
SD	83/66	13.6	0.30	0.20-0.45	**<0.0001**	0.36	0.23-0.56	**<0.0001**
CR/PR	30/23	19.53	0.14	0.08-0.25	**<0.0001**	0.18	0.10-0.32	**<0.0001**
PD	46/43	5.33	Ref			Ref		
**Subsequent line** (n=178)								
No	105/83	8.37	Ref			Ref		
Yes	73/66	14.67	0.54	0.39-0.75	**<0.0001**	0.48	0.33-0.70	**<0.0001**

* Statistically significant; *P* value <0.05

Abbreviations: ULN, upper limits of normal; PFS, Progression-free survival; OS, overall survival; SD, stable disease; CR, complete response; PR, partial response; PD, progressive disease

Supplementary Table 3. Treatment-related adverse events

**Adverse events**<br>*All grades*<br>**Grade 3 or Higher**	**First-line chemotherapy**				***P****
	**Gemcitabine**<br>n (%)	**Gemcitabine & capecitabine**<br>n (%)	**(m)FOLFIRINOX**<br>n (%)	**Platinum doublets**<br>n (%)	
**Anemia**	*66(60)*<br>**16(14.5)**	*56(53.5)*<br>**11(12.8)**	*53(59.6)*<br>**24(27)**	*20(66.7)*<br>**7(23.3)**	*0.20*<br>**0.05***
**Neutropenia**	*60(54.5)*<br>**15(13.6)**	*41(47.7)*<br>**13(15.1)**	*18(20.2)*<br>**11(12.4)**	*15(50)*<br>**2(6.7)**	*<0.0001**<br>**0.67**
**Febrile neutropenia**	*0(0)*	*4(4.7)*	*5(5.6)*	*0(0)*	*0.06*
**Thrombocytopenia**	*21(19.1)*<br>**6(5.5)**	*11(12.8)*<br>**3(3.5)**	*26(29.2)*<br>**6(6.7)**	*7(23.3)*<br>**2(6.7)**	*0.33*<br>**0.78**
**Neuropathy**	*0(0)*<br>**0(0)**	*1(1.2)*<br>**0(0)**	*28(31.5)*<br>**4(4.5)**	*7(23.3)*<br>**0(0)**	*<0.0001**<br>**0.02***
**Diarrhea**	*5(4.5)*<br>**3(2.7)**	*18(20.9)*<br>**3(3.5)**	*28(31.5)*<br>**10(11.2)**	*2(6.7)*<br>**0(0)**	*<0.0001**<br>**0.02***
**Palmar-plantar erythrodysesthesia**	*0(0)*<br>**0(0)**	*30(34.9)*<br>**7(8.1)**	*0(0)*<br>**0(0)**	*3(10)*<br>**1(3.3)**	*<0.0001**<br>**0.001***

*Adverse events of all grades are indicated in *italic text*, while those classified as Grade 3 or higher are presented in **bold text.**

* Statistically significant; *P* value <0.05
